# The Age of Brain Organoids: Tailoring Cell Identity and Functionality for Normal Brain Development and Disease Modeling

**DOI:** 10.3389/fnins.2021.674563

**Published:** 2021-08-13

**Authors:** Lisiane O. Porciúncula, Livia Goto-Silva, Pitia F. Ledur, Stevens K. Rehen

**Affiliations:** ^1^Department of Biochemistry, Program of Biological Sciences - Biochemistry, Institute of Health and Basic Sciences, Federal University of Rio Grande do Sul (UFRGS), Porto Alegre, Brazil; ^2^D'Or Institute for Research and Education (IDOR), Rio de Janeiro, Brazil; ^3^Department of Genetics, Institute of Biology, Federal University of Rio de Janeiro (UFRJ), Rio de Janeiro, Brazil

**Keywords:** brain organoids, brain development, neurodevelopmental disorders, electrophysiology, human pluripotent stem cells (hPSC), neurodegenerative diseases, Zika virus, SARS-CoV-2

## Abstract

Over the past years, brain development has been investigated in rodent models, which were particularly relevant to establish the role of specific genes in this process. However, the cytoarchitectonic features, which determine neuronal network formation complexity, are unique to humans. This implies that the developmental program of the human brain and neurological disorders can only partly be reproduced in rodents. Advancement in the study of the human brain surged with cultures of human brain tissue in the lab, generated from induced pluripotent cells reprogrammed from human somatic tissue. These cultures, termed brain organoids, offer an invaluable model for the study of the human brain. Brain organoids reproduce the cytoarchitecture of the cortex and can develop multiple brain regions and cell types. Integration of functional activity of neural cells within brain organoids with genetic, cellular, and morphological data in a comprehensive model for human development and disease is key to advance in the field. Because the functional activity of neural cells within brain organoids relies on cell repertoire and time in culture, here, we review data supporting the gradual formation of complex neural networks in light of cell maturity within brain organoids. In this context, we discuss how the technology behind brain organoids brought advances in understanding neurodevelopmental, pathogen-induced, and neurodegenerative diseases.

## Introduction

Given the limited accessibility to human brain tissue, most of the knowledge about brain development comes from studies in animals. Contributions include genetic manipulation *in vivo*, which allowed the discovery of key genes regulating neurodevelopment (Gavériaux-Ruff and Kieffer, 2007). However, from an evolutionary perspective, rodents, in particular, cannot reproduce the cortical regionalization and cytoarchitectonic fields that are unique to the human brain (Clowry et al., 2010; Molnár and Clowry, 2012).

Brain organoids have been referred to as cellular aggregates resembling brain regions under development. They allowed for a better comprehension of cell division processes in the human brain. For the first time, it was possible to verify that cell divisions within the ventricular zone (VZ) of this live model of the human brain are mostly symmetrical. This is in contrast to the observed in the mouse brain, which presents mixed symmetrical/asymmetrical divisions. This difference may explain the increased size of the human cortex in comparison to the mouse cortex. Also, genetic mutations that cause microcephaly in humans do not produce similar effects in the mouse brain, demonstrating the fine regulation in the growth of the cortex during evolution (Gabriel et al., 2020).

Human brain organoids can be generated by non-guided protocols in which the neural tissue develops from intrinsic cues. This results in brain organoids displaying domains that resemble multiple brain regions (Lancaster et al., 2013; Quadrato et al., 2017). Guided protocols can be used to generate brain organoids that resemble specific brain regions, such as “forebrain organoids,” “midbrain organoids,” and “hindbrain organoids” [Kadoshima et al., 2013; reviewed in Xiang et al. (2021)]. Besides that, many efforts have been made to model inter-regional interactions by fusing brain organoids with characteristics of distinct brain regions, which were termed as “assembloids” (Bagley et al., 2017; Birey et al., 2017; Xiang et al., 2017). Advances in the field also led to the optimization of organoid cultivation to circumvent low reproducibility, inter-batch variability and to offer better conditions for recapitulating the tridimensional architecture of complex tissues [reviewed in Del Dosso et al. (2020); Tanaka et al. (2020)].

As brain organoids allow for the study of multiple neural cell types and their interactions, we will focus this review on some aspects of their mature neural cells.

By presenting the evidence for specific cell types across non-guided and guided protocols, we aim to cover the main findings regarding organoid physiology over time in culture. Besides, we will also discuss studies pointing to the potential of cerebral organoids (COs) to model neurological disorders. The idea is to paint a picture of organoid functionality based on maturity and how that can be applied in the context of disease modeling.

## Cell Identity in Brain Organoids

**Figure 1:**
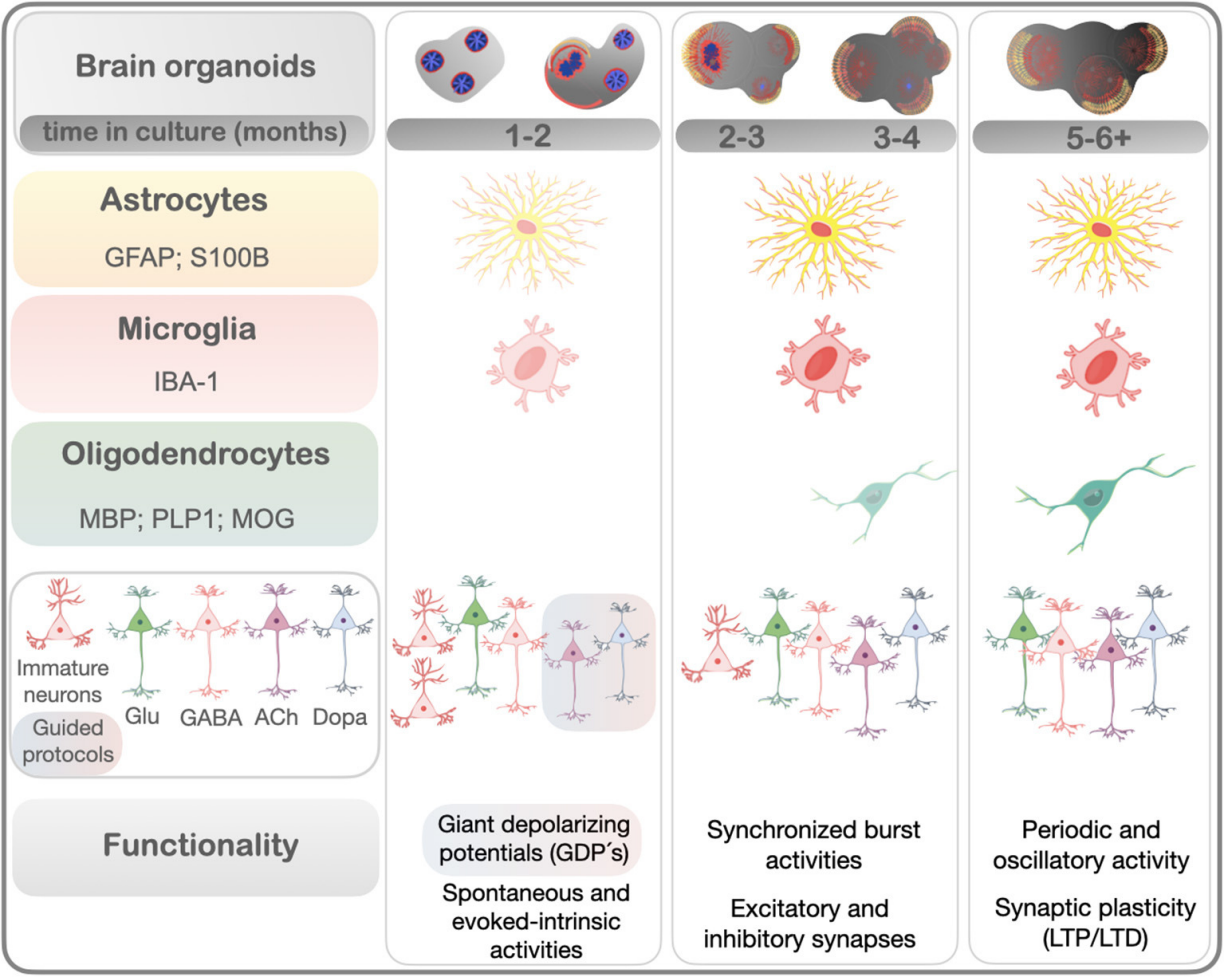
Cell types/markers and functional properties reported in brain organoids in a time-dependent fashion, according to the literature. This timeline summarizes the emergence of the main mature cell types in brain organoids from distinct protocols, shown in months. Neurons start to appear in earlier times of culturing (~1 month). Dopaminergic and cholinergic neurons were found enriched in guided protocols. Markers of astrocytes and microglia increase from 1 month, while oligodendrocytes gradually emerge from 3 months of culturing. Early functional properties of developing brains such as giant depolarizing potentials (GDPs)-like can be recorded around 1 month of culture in guided protocols. Electrophysiological maturation such as synchronized burst firing activities, both action and spontaneous inhibitory/excitatory postsynaptic potentials, gradually emerge over time (from 2 months). More complex neural networks resulting from interconnectivity were reported by the presence of periodic oscillatory activity and synaptic plasticity and included long-term potentiation and depression (LTP and LTD) (~5–6 months). Neurons: Glu - glutamatergic; GABA, GABAergic; Dopa, dopaminergic; ACh, cholinergic.

Distinct cell types represented in a human brain organoid could be addressed by proteomic and transcriptomic analyses (Quadrato et al., 2017; Goto-Silva et al., 2019; Nascimento et al., 2019). As the tridimensional (3D) structures of brain organoids develop, different cell types proliferate, migrate, and differentiate. Astrocytes, neurons, oligodendrocytes, microglia, and even the re-construction of vasculatures have been generated in brain organoids (Lancaster et al., 2013; Di Lullo and Kriegstein, 2017; Gabriel and Gopalakrishnan, 2017), within different times in culture (Figure 1, standardized in months). In this section we dissect how these cell types appear and mature in brain organoids under established protocols (reviewed in Del Dosso et al., 2020; Sidhaye and Knoblich, 2021).

### Glutamatergic Neurons

#### Evidence From Non-guided Protocols

Evidence of glutamatergic cells was reported in non-guided protocols of cerebral organoids (COs) (2.5 months) when frequent calcium spikes were observed after the addition of exogenous glutamate (Lancaster et al., 2013). In 3-month-old cerebral organoids, excitatory cortical neurons comprised almost 50% of the cells, shifting to ~23% after 4 months (Fair et al., 2020). Glutamatergic synapses were identified in 6-month-old human cortical spheroids by the co-localization of either vGluT-1 with synapsin (SYN-1) or NMDA receptor subunit NR2B with postsynaptic protein PSD-95 (PSD-95) visualized by array tomography. These cortical glutamatergic neurons displayed a layer-specific organization (Paşca et al., 2015). The presence of glutamatergic synapses was confirmed by the co-localization of pre and postsynaptic markers VGluT1 and PSD95 in dorsal forebrain organoids with 3- and 6-months (Velasco et al., 2019).

#### Evidence From Guided Protocols

In guided protocols, glutamatergic neurons (positive for GLUD1, vGLUT1, and vGLUT2) were highly enriched in 1-month brain organoids resembling cortical domain (Xiang et al., 2017). Similarly, glutamatergic neurons (vGluT1) were detected in 1-month cortical organoids, reaching a substantial portion of the neuronal population between 3 and 6 months in culture, with some of them co-expressing subunits of GABAergic receptors (Trujillo et al., 2019). Similar to GABA transcripts, glutamatergic markers were highly expressed in 1–1.3 month Bioengineered Neuronal Organoids (BENO's) as well as cortical neurons positive for vesicular glutamate transporter (vGLUT) and receptor (GLUR1) (Zafeiriou et al., 2020).

Single-cell profiling analysis revealed that glutamatergic genes were also highly expressed after 2 months of culturing of human subpallium spheroids (Birey et al., 2017), with a predominance of 55% of the total cells in 3.5-month human cortical spheroids encompassing glutamatergic neurons co-expressing cortical layer markers (Yoon et al., 2019). A small group of glutamatergic neurons, identified by genes encoding glutamate transporters (SLC17A7 and SLC17A6), was identified in 2.5-month striatal spheroids, but their expression was more correlated with the neocortex and amygdala (Miura et al., 2020). Overall, glutamatergic neurons have been abundantly observed in cortical organoids.

### GABAergic Neurons

#### Evidence From Non-guided Protocols

Calretinin-positive cells have been observed among neuronal types found in self-organized COs (obtained with non-guided protocols which did not display the identity of a specific brain region). They have a typical morphology of tangential migration neurons that is suggestive of interneurons. Also, GABA positive (vGAT) cells were observed in regions resembling medial ganglionic eminence (MGE) (Lancaster et al., 2013; Renner et al., 2017). Immunohistological analysis in 3-month-old forebrain organoids showed GABAergic neurons expressing VGAT, parvalbumin, nNOS, or somatostatin (Qian et al., 2016). By culturing slices from COs in an air-liquid interface, named (ALI-CO), Giandomenico and collaborators showed an improvement in cell viability and maturation when compared to previous non-guided protocols. This protocol also led to higher levels of GABA and expression of inhibitory synapse formation genes in 2.5 months-old ALI-CO (Giandomenico et al., 2019). In a recent study, single-cell RNA sequencing comparison of COs at two time points found 1.8% of cells to be interneurons after 3 months in culture—a number that reached 30.4% at 4.3 months. Besides, the expression of GABA (parvalbumin or somatostatin) for mature GABAergic neurons increased in cerebral organoids and acquired a mature phenotype (larger cell bodies) between 3 and 5 months in culture (Fair et al., 2020). A similar analysis had been performed in non-guided COs that identified the expression of both cortical GABAergic interneuron genes (including markers DLX5, DLX2, SCGN, and GAD1) and genes of GABAergic synapses by 6-months in culture (Quadrato et al., 2017). Even though GABAergic interneurons originate from ventral forebrain progenitors, some GABA genes and markers may be found in cerebral organoids, whose cell diversity encompasses mainly regions of the rostral forebrain.

#### Evidence From Guided Protocols

GABAergic neurons were observed in brain organoids exposed to dorsal patterning clues around 2.5 months of culturing (Xiang et al., 2017). In a more detailed analysis of dorsal spheroids, including bulk RNA sequencing, a small proportion of GABAergic neurons was found in human cortical spheroids at 3.5 months, which may be assigned to a minor ventralization that has eventually occurred (Yoon et al., 2019). Trujillo et al. used guided protocols, specific to generate region-specific organoids, with cortical specification (Paşca et al., 2015; Yoon et al., 2019). They showed that human cortical organoids should be cultured for 3–6 months before GABAergic neurons could be observed, and for 6 months to detect GABA in the culture medium. Even after long periods of maturation (up to 10 months), GABAergic neurons comprised only 15% of the total population of neurons (Trujillo et al., 2019). Thus, the presence of GABAergic markers occurs to a lesser extent in organoids with a dorsal domain and requires more time of culture, as a predictable pattern resembling human brain development.

Studies comparing different species showed that 40% of supragranular layers, among all cortical neurons, were identified as interneurons in primates vs. ~15–20% in rodents (Defelipe, 2011). Likewise, 5% of neurons are interneurons in the mouse thalamus, while in humans, they represent 30% (Arcelli et al., 1997). Since interneurons mainly migrate from the ventral telencephalon, some groups aiming to model interneuron migration have developed protocols guided to either the ventral (subpallium) or dorsal (pallium) forebrain. *In vivo*, the ganglionic eminences (GE) generate interneurons that migrate tangentially into the dorsal forebrain (Hansen et al., 2013; Ma et al., 2013). As a result, an enrichment of GABAergic markers was identified according to the specification of brain regions in a shorter time in culture. In brain organoids of the medial ganglionic eminence, the expression of GABA synthesis enzymes and vesicular GABA transporter (vGAT) genes were highly enriched after 1 month, while GABAergic synapses were confirmed by immunohistochemistry after 2 months in culture (Xiang et al., 2017). Likewise, in human subpallium spheroids, GAD67 (GABA enzyme synthesis), somatostatin, calretinin, calbindin, and GABA *per se* were already observed within 2 months. Single-cell profiling identified a group of GABAergic cells (DLX1, GAD1, SLC32A1, SCG2, SST) at 3.5 months, while mature cells positive for parvalbumin were described only after 6 months of culturing (Birey et al., 2017). The fusion of ventral and dorsal cerebral organoids presented GAD1 or vGAT positive cells in both regions at 2.6 months and were dorsally stained for GAD1 cells migrating from the ventral region (Bagley et al., 2017).

To model cortico-striatal circuits of the forebrain, spheroids resembling the lateral ganglionic eminence were differentiated to give rise to human striatal spheroids. Single-cell RNA-sequencing analyses identified more than 50% GABAergic genes (STMN2+, SYT1+) and GABA-synthesizing enzymes (GAD1 and GAD2) in these organoids at 2.5 months. Immunostaining for calbindin, GAD67, and other markers indicated the presence of medium spiny neurons in the striatal spheroid (Miura et al., 2020). In human midbrain-like organoids, GABA-positive mature neurons were detected after 1 month in culture (Jo et al., 2016), similar to the observed in BENOs, which showed transcripts of GABA as well as cells positive for GABA receptors around 1 month in culture (Zafeiriou et al., 2020).

### Dopaminergic and Cholinergic Neurons

Extensive research has focused on generating dopaminergic and cholinergic neurons *in vitro*. Since from all neurons identified in rodent striatum primary cultures, only 3% are dopaminergic neurons, the neuroblastoma cell line (SH-SY5Y) has been extensively used as neuron-like cells. Under differentiation, these cells usually do not express the axonal component, but dendritic and either dopaminergic or cholinergic markers are abundantly expressed (Lopes et al., 2010; de Medeiros et al., 2019). Although a relatively high amount of striatal neurons were obtained from human pluripotent stem cells, they were mostly immature (Delli Carri et al., 2013; Arber et al., 2015).

Six-month-old COs present molecular signatures of dopaminergic neurons, expressing tyrosine hydroxylase and EBF1, and immunohistological analysis found neurons positive for tyrosine hydroxylase from 1 to 9 months (Quadrato et al., 2017). A previous study showed that 1–2 month midbrain organoids presented either tyrosine hydroxylase or dopamine transporter positive neurons co-expressing FOXA2 (floor-plate precursor marker). After 2 months, midbrain organoids were dissociated, and 95% of cells cultured as a monolayer were FOXA2 positive, with 55% of them co-expressing tyrosine hydroxylase (Qian et al., 2016).

Further study with the generation of human midbrain-like organoids and human striatal spheroids showed that, after 1 month in culture, neuroectoderm toward the floor plate in human midbrain-like organoids was similar to the midbrain *in vivo*. After 1.5 months, 54% of neuronal progenitors found in midbrain-like organoids co-expressed tyrosine hydroxylase. At 2 months, 22% of all mature neurons co-expressed tyrosine hydroxylase, and 30% expressed dopamine transporter. Furthermore, dopamine measurements within human midbrain-like organoids gradually increased as they matured (Jo et al., 2016). Similarly, ~30% of mature neurons expressed dopamine- and cAMP-regulated phosphoprotein 32 (DARPP32) in 2.5–3-month striatal spheroids, in which both dopamine receptors DRD1 and DRD2 genes were identified. D2 receptors were visualized after 4 months (Miura et al., 2020).

While cholinergic markers had not been detected in human cortical spheroids, cholinergic motor neurons were identified in human spinal cord spheroids by immunostaining for the enzyme that catalyzes the biosynthesis of the acetylcholine (CHAT) and the vesicular acetylcholine transporter VACHT after 1 month in culture (Andersen et al., 2020). BENOs with similar time in culture expressed various cholinergic transcripts, like CHAT, ACHE, and SLC18A3 (Zafeiriou et al., 2020). In human subpallium spheroids, dopaminergic and cholinergic genes were highly expressed after 2 months (Birey et al., 2017).

### Astrocytes

Astrocytes actively participate in synaptic pruning during development and are essential players in synapse formation and maturity of neural networks (Chung et al., 2013). Despite some intrinsic limitations that include maintaining long-term cultures, we and others have generated astrocytes directly from iPSCs and explored their functional properties (Zhou et al., 2019; Trindade et al., 2020). In brain organoid models, radial glia and astrocytes have been previously described. Nascimento and collaborators performed proteomics analysis in non-guided COs (1.5 months) and found the expression of proteins like GFAP, VIM, GMFB, and ALDH1L1 (Nascimento et al., 2019). As both radial glia and mature astrocytes can be identified by the expression of GFAP, astrocyte maturity is often understated. Pasca et al. observed about 3% of GFAP positive cells over the 1st month of differentiation in a human cortical spheroid model. This number increased to ~8% by 2.5 months in culture and to almost 20% by 6 months in culture. After almost 2 months, thin GFAP positive processes were already observed in the cortical spheroid parenchyma intermingled with NEUN positive cells (Paşca et al., 2015). Qian et al. observed S100β and GFAP expression in astrocytes that were in close proximity to surrounding neurons in 3.3-month-old forebrain organoids (Qian et al., 2016). In 2017, Renner et al. found strong GFAP staining and astrocytic morphology in a non-guided protocol, starting at 3 months in culture and increasing with time in culture up to 7 months. Interestingly, these organoid astrocytes exhibited features of reactive astrocytes, particularly along the surface (Renner et al., 2017). Quadrato et al. (2017), have used single-cell RNA sequencing to profile over 80 thousand cells from 31 COs at 3 and 6 months *in vitro*. Over 8 thousand of the cells analyzed expressed genes of adult human astrocytes, like AQP4 and GFAP. Mature astrocyte markers were only present at 6-month old organoids (Quadrato et al., 2017). Yoon and collaborators found a progressive increase in GFAP expression over 6 months *in vitro* in cortical spheroids, representing more than 15% of the total cell number (Yoon et al., 2019). In 3-month cultures of a dorsal forebrain organoid, astroglial cells comprised about 13% of the total cell number. However, at 6 months, 53.5% of the cells were considered mature astrocytes by the expression of GFAP and S100B (Velasco et al., 2019). Trujillo and collaborators claimed that cortical organoids comprised mainly glia at 3 and 6 months, characterized by the expression of SLC1A3, with <5% of GFAP+ cells. However, the authors noted an increase in the GFAP+ population to about 30–40% after 6 months of differentiation (Trujillo et al., 2019). Marton and collaborators found 8% of the cells to be GFAP positive in their human oligodendrocyte spheroids. This number increased to 21% around 3.5 months of differentiation (Marton et al., 2019). Bioengineered Neuronal Organoids (BENOs) were also enriched in astrocyte markers with time in culture, and GFAP/S100beta expression increased after 2 months of differentiation (Zafeiriou et al., 2020). Fair and colleagues also observed an increase in GFAP+ and S100b+ astrocytes by 5 months in culture, with processes that were spread and ramified, features that indicate maturation as well as association with neuronal populations (Fair et al., 2020). A developmental time course for GFAP+ cells noted a progressive increase from <2.5% in dissociated human cortical spheroids at 1.6 months to about 20% at 4.3 months in culture (Yoon et al., 2019). GFAP positive cells were observed in 2.5-month-old human spinal cord spheroids that also expressed MBP (Andersen et al., 2020).

Moreover, astrocyte maturity in cerebral organoids has been thoroughly investigated by transcriptomic, functional, and proteomic analysis, confirming that these cells are functionally identical to astrocytes isolated from adult human brains (Dezonne et al., 2017; Sloan et al., 2017). Regarding functionality, features that distinguish immature from mature astrocytes include participation in synapse formation, neuronal calcium signaling, glutamate uptake, and phagocytosis, which are crucial for synaptic pruning (Sloan et al., 2017). These properties were evaluated in astrocytes obtained by immunopanning of 5–14-month human cortical spheroids. The authors noted glutamate uptake, synaptogenic properties, and a decline of synaptic phagocytic activity, as well as an increase of neuronal calcium responses that were time-dependent (Sloan et al., 2017).

### Oligodendrocytes

A model with myelinated oligodendrocytes is essential for exploring electrophysiology, myelination, and interactions of oligodendrocytes with neurons and astrocytes; therefore, it is fundamental for modeling human diseases (Madhavan et al., 2018; Marton et al., 2019). By month 3.5 in culture, neurocortical spheroids normally have a good portion of neurons and astrocytes but no oligodendrocytes (Madhavan et al., 2018). Moreover, non-guided protocols usually fail to originate cells from the oligodendrocytic lineage (Tanaka et al., 2020). However, at least two research groups described the presence of oligodendrocytic factors in non-guided protocols, detected through proteomics and RNA seq. Nascimento and collaborators found proteins related to oligodendrocyte precursor cells (OPCs) and more mature oligodendrocyte transitional stages in 1.5-month old COs, analyzed by mass spectrometry-based proteomics. Proteins related to the OPC stage included CNP and PDGFRA, while proteins related to oligodendrocytes comprised MBP, PLP1, and MOG. Moreover, the authors observed early stages of lamellae formation under electron microscopy in these young organoids and were able to isolate OPCs from 1.5-month old organoids (Nascimento et al., 2019). Quadrato et al. also found clusters of cells that expressed myelinating factors APOD and CSPG4 (Quadrato et al., 2017). In order to enrich the population of oligo cells in organoids, Madhavan et al. described a protocol in 2018 to generate oligodendrocyte precursor cells (OPCs) and myelinating oligodendrocytes named oligocortical spheroids. To obtain these cells, the authors treated 1.6-month (50-day) cortical spheroids with PDGF-AA (platelet-derived growth factor AA) and IGF-1 (insulin-like growth factor 1), known to drive the expansion of OPCs, for 10 days. Between 1.6–2 months, native OPCs could be observed. Then, T3 thyroid hormone was added to induce oligodendrocyte differentiation and myelination, between 2 and 2.3 months (60–70 days). The treatment periods mimic the initial specifications of OPCs and oligodendrocytes in the human fetal brain at weeks 10 and 14 after conception. The authors observed multiple layers of uncompacted myelin wrapping axons under electron microscopy, but no evidence of structural organization as late as month 7.5 (30 weeks) (Madhavan et al., 2018). A year later, Marton et al. developed a protocol to generate human oligodendrocyte spheroids. This protocol differed from the one from Madhavan et al., as the addition of oligo-specific growth factors happened sooner, at day 25. The spheroid culture expressed forebrain markers NKX2-1, OTX2, PAX6, LHX2, SIX3, and FOXG1 at similar levels to human cortical spheroids at day 37. After 3.3 months (100 days) in culture, the authors found a significant increase in the expression levels of OLIG2, MBP, and NKX2-2 when compared with human cortical spheroids. At months 1.8 and 3.6 (days 54 and 110), 12 and 9% of the cells were double positive for NKX2-2 and OLIG2, respectively, with 51 and 35% being PDGFα positive. The authors also found a range of pre-oligodendrocytes to late-stage mature oligodendrocytes at months 3.3 and 5.3 (days 100–160) of differentiation, with O4, O1, and MBP positive cells. Their methodology was capable of producing oligodendrocytes in proximity to neurons and astrocytes. Moreover, the authors found that the OPCs in human oligodendrocyte spheroids migrated, with a peak between 3.6 and 6 months (days 110 and 180) of *in vitro* culture. At 5–5.3 months (days 150–160), about 28% of oligodendrocytes (MBP+) interacted with NF-H+ processes, and different stages of myelination were observed under electron microscopy (Marton et al., 2019).

While Andersen and collaborators observed the expression of MBP by immunohistochemistry in a human spinal cord spheroid model after 2.5 months (Andersen et al., 2020), Xiang and collaborators developed an organoid system to model human medial ganglionic eminence (MGE) development, a region known as the main origin of cortical interneurons and related lineages. In this work, MGE organoids were merged to cerebral organoids to mimic the migration of interneurons to the cortex. The authors confirmed immunostaining for OLIG1 in MGE organoids with almost 3 months (81 days, to be precise), suggesting that oligodendrocyte genesis from MGE precedes that of the cortex (Xiang et al., 2017). In bioengineered neuronal organoids (BENOs), the first oligodendrocyte progenitors (OLIG2+ cells) were detected in 2-month organoids. After 3 months in culture, the first myelinated axons were observed, together with MBP and CNP expressions. By 5 months in culture, the number of CNP and OLIG2 positive cells, together with myelinated axons, increased substantially, as observed by immunofluorescence (Zafeiriou et al., 2020).

### Microglia

Microglia are the primary innate immune cells of the brain. The importance of these cells during brain development resides in their role on the release of diffusible factors and phagocytosis supporting synaptic pruning (Stevens et al., 2007; Paolicelli et al., 2011). Even though microglia are derived from mesodermal cells, no substantial expression of these cell types has been detected in brain organoids. For example, in the work from Quadrato et al. (2017), the authors identified a cluster of mesodermal cells by single-cell RNA sequencing. By using the same brain organoid protocol as Quadrato et al. (2017) with minor modifications, Ormel et al. were able to identify microglia-like cells that developed from the mesodermal progenitors. IBA-1 positive cells were low in numbers in organoids about 1 month (24 days) but increased at about 2 months in culture (52 days), spreading throughout the organoid. Microglia cells generated within the organoid also expressed other microglia-specific genes like nuclear marker PU.1, as well as CD68, RUNX1, SPI1, CD68, IRF8, TGFBR1, CSF1R, and others (Ormel et al., 2018).

### Endothelial Cells

While microglial cells were shown to originate spontaneously from non-guided protocols for the generation of COs, the same was not true for endothelial cells. Studies with clusters that showed endothelial gene signatures with high expression have failed to report any evidence of vascular tissue. To circumvent this, different protocols were created. Pham and collaborators differentiated iPSCs from the same patient into endothelial cells while growing them into brain organoids. When the organoid was about a month old, it was re-embedded in matrigel with the endothelial cells, growing *in vitro* for another 3–5 weeks or *in vivo*. The authors found CD31 positive cells around the organoid as well as capillaries and tubular structures that originated from the endothelial cells (Pham et al., 2018). Another protocol engineered human embryonic stem cells (hESCs) to express ETV2, a transcription factor shown to induce the differentiation of endothelial cells. These organoids formed a vascular-like network that improved the maturation of the brain organoid, including the presence of blood-brain barrier features (Cakir et al., 2019).

## Structural and Functional Maturity

### Neurons

The formation of neural networks wisely obeys certain rules and the hierarchical organization of neuronal assemblies. Therefore, the complexity in neural networks coincides with increased synapse formation. How long do brain organoids need to mature to offer insights about synaptic functioning? Recent studies have shown that brain organoids are able to recapitulate some features of human brain neuronal network formation (Passaro and Stice, 2020), with hierarchical spatial and functional organization according to adopted protocols and their time in culture (Figure 1).

Insights on the functionality of brain organoids were initially performed in slices from 4-month human cortical spheroids and dissociated cultured neurons. After 2 weeks in culture, neurons showed action potentials, inward Na+ current blocked by tetrodotoxin, followed by activation of a more sustained K+ current and abundant spontaneous calcium spikes. In slices, network activity was detected by fire action potentials in response to depolarizing current steps, large-amplitude excitatory postsynaptic potentials (EPSPs) after extracellular electrical stimulation, spontaneous and stimulated spiking, and burst events (Paşca et al., 2015). In a further study, spontaneously firing neurons were detected in 8-month COs (but not 4-month ones). At the same time, neuronal networks were characterized by neurons exhibiting coordinated activity with temporal organization for neuronal recruitment and firing and a subpopulation of photosensitive retinal-like cells responsive to light stimulation (Quadrato et al., 2017).

In order to evaluate neural network formation, cells were dissociated from 2 to 3.5-month COs and cultivated for different time points (up to 12 months). While organoids at 2.5–3.5 months showed spontaneous and asynchronous calcium transients, suggestive of an immature network, cultured neurons showed a synchronized pattern for calcium transients together with bursts in response to glutamate, which was blocked by GABA and glutamate antagonists (Sakaguchi et al., 2019). In 4-month-old ALI-COs the functionality of mature neuronal architectures, which included axon bundles with typical tract morphology, was confirmed by network bursts with neurons showing simultaneous bursts of action potentials. A degree of spatial specificity was observed in the connections within ALI-COs (Giandomenico et al., 2019).

In addition to action potentials and voltage-dependent Na+ current blocked by tetrodotoxin, the functionality of excitatory neurons in 6-month cortical organoids was evidenced by the blockade of spontaneous excitatory postsynaptic currents with glutamate antagonists. In the same work, various patterns of synchronic network activity were registered in 6–10 months of culturing and the oscillatory network activity was also reported as similar to those observed in preterm infant electroencephalograms (Trujillo et al., 2019). Likewise, the electrophysiological maturation of COs was also tracked at different points by probing them with microelectrode array recordings every month in culture. COs showed a significant and gradual increase of spontaneous electrical activity from 1 month up to 5 months in culture, but functional maturity was characterized by network bursting events and also synchronized burst firings observed from 4 to 5 months (Fair et al., 2020).

Guided protocols have also been studied regarding their network maturity activity of brain organoids. Action potentials and excitatory postsynaptic currents were detected in neurons within 3–4 month spheroids of dorsal forebrain, while ~75% of neurons from spheroids of ventral forebrain exhibited inhibitory postsynaptic currents, which were abolished by GABA_A_ antagonist (Birey et al., 2017). Neurons within human midbrain-like organoids from 1 to 2 months of culture showed action potentials, fast and inactivating inward Na+ and outward K+ currents. In addition, decreased resistance and increased membrane capacitance, as well as spontaneous excitatory and inhibitory postsynaptic currents were considered typical features of mature neurons. Network activity was evidenced by large-amplitude excitatory postsynaptic potentials in response to extracellular electrical stimulation. Importantly, dopaminergic neurons (20%) were functionally identified by rhythmic discharges, rebound depolarizations, and suppression of firing after D2/D3 receptor agonist application (Jo et al., 2016).

In slices obtained from human striatal spheroids, electrophysiological features of striatal medium spiny neurons were time-dependent. While inward rectification was observed at 4 months, slow-ramp depolarization with delayed first spike, and hyperpolarization of the resting potential were detected in 15% of human striatal spheroid neurons at 5 months of culturing (Miura et al., 2020).

Neuronal excitability comprises the balance between glutamatergic excitatory input and GABAergic inhibitory transmission. Different from mature neurons, GABA acts as an excitatory neurotransmitter in immature neurons due to higher intracellular Cl–concentration, triggering sodium spikes. The GABAA-activated Ca2+ influx regulates the expression of the chloride extruder KCC2 (Ganguly et al., 2001; Ben-Ari et al., 2007). It is now established that GABA_A_ is responsible for excitatory neurotransmission during early periods of brain development and shifts its action to inhibitory after a gradual reduction of intracellular chloride levels, mediated by NKCC1 down-regulation and KCC2 up-regulation (Cherubini et al., 1991; Rivera et al., 1999). Interestingly, forebrain organoids expressed NKCC1 between 1.5 and ~3 months, whereas KCC2 was strongly expressed after 2.5 months. In that time, the authors monitored Ca2+ rise in response to GABA-induced depolarization. They were able to show that among all neurons that responded to glutamate, the percentage of neurons without GABA-induced Ca2+ rise increased over time (Qian et al., 2016).

Importantly, even though there is a small number of functional synapses during brain development, immature neurons present a high degree of synchrony that can be assessed. Most of the synaptic activity is provided by a primitive form of network-driven activity characterized by giant depolarizing potentials (GDPs), which are mediated by depolarizing GABAergic responses typically found in immature neurons (Ben-Ari, 2002; Ben-Ari et al., 2012). In rodents as well as in humans, GDP frequency initially increases during fetal development, then it decreases, and then it ceases completely in early postnatal life (Corlew et al., 2004; Moore et al., 2011). Recently, Bioengineered Neuronal Organoids (BENOs) developed three distinct stages of network development, enabling the authors to show, among others, GDP-like events by 1 month (Figure 1). Their incidence gradually reduced after 1.3–2 months and was modulated by GABA and glutamate antagonists in a time-dependent manner, which revealed a developmental switch of GABA from excitatory to inhibitory around 1.3 months. In parallel, features of spatiotemporal neuronal network organization revealed increased individual and network bursts (NB), bursts organized in complex synchronous events, and synchronous firing over time in culture, peaking at 2 months. In addition, evidence for paired-pulse depression and short- and long-term potentiation and depression (STP/D and LTD/P) were observed after 1.3 months of culture. Overall, these data showed that BENOs formed complex neuronal networks with shorter culture time (Zafeiriou et al., 2020). These findings were particularly relevant when one considers that organoids may contain structures and spatial organization that recapitulate the functionality of human brain regions during early periods of their development and also neuronal plasticity.

Even though gaining access to a more complex functional integration of microcircuits is challenging, advances in the assembloid methodology have provided recordings of functional neural circuits in a more sophisticated way. Owing to the need to assess neural projections extension, interneurons migration and integration, the dorsal-ventral forebrain axis was recreated and tracked with fluorescent reporters. Meanwhile, well-documented cell migration has been observed in assembloids (Bagley et al., 2017; Birey et al., 2017; Xiang et al., 2017), with functionality evaluated before and after fusing them (Birey et al., 2017; Xiang et al., 2017).

In whole-medial ganglionic eminence organoids, action potentials and calcium transients (blocked by tetrodotoxin) were detected around 1–2 months of culture, and synchronized calcium activities enhanced after adding GABA_A_ antagonist. After assembling medial ganglionic eminence and cortical organoids, calcium transients were detected in cells from the cortical side, and neurons from the medial ganglionic eminence organoid side were identified as GABAergic after migration (Xiang et al., 2017). Besides, IPSCs were detected in neurons from the dorsal forebrain side (before fusion, only excitatory postsynaptic currents were present). After electrical stimulation, evoked excitatory postsynaptic currents started to be detected in fused slices, and multisynaptic IPSCs were sensitive to GABA_A_ antagonist, which demonstrate functional integration after neuronal migration (Birey et al., 2017).

In a recent study, Andersen and collaborators showed cortico-spinal connections when combining cortico-spinal organoids with iPSC-derived skeletal muscle organoids. After optogenetic stimulation, cortical organoids were able to contract skeletal muscle organoids (Andersen et al., 2020). With another approach mentioned above, ALI-COs developed tracts, which included escaping extracortical projections that are able to innervate mouse embryo spinal cord cultures and trigger muscle contraction (Giandomenico et al., 2019).

### Glial Cells

Glial cells can also be assessed regarding their functionality. In a previous study, more synaptic induction was observed when retinal ganglion cells were cultured beneath human cortical spheroid-derived astrocyte lineage cells. Besides, compared to earlier time points, dissociated neurons from ~3-month old human cortical spheroids increased peak [Ca2+]i amplitude after depolarization when co-cultured with astrocytes isolated from human cortical spheroids at 12–14.5 months of culture (Sloan et al., 2017). Calcium imaging revealed that cultured astrocytes derived from 1.5-month COs were responsive to ATP and showed typical asynchrony features (Dezonne et al., 2017).

Marton et al. identified the expression of GABAergic-related gene GAD1 more abundantly in 3.3-month old human oligodendrocyte spheroids than the expression of glutamatergic-transporter-encoding SLC17A7 gene (VGLUT1). OPCs in these oligodendrocyte spheroids expressed voltage-dependent sodium and potassium channels from about 3.6–5.8 months in culture that were not observed in multipolar cells, an expected progression as OPCs mature. Potassium currents seemed to be inactivated slowly, with complementary differences in input capacitance and resistance. Cells of the oligodendrocytic lineage were sensitive to glutamate release, an indication that they express ionotropic glutamate receptors that are activated in human oligodendrocyte spheroids (Marton et al., 2019).

Collectively, data reviewed here shows that the formation of complex networks in brain organoids takes time and depends on the abundance of synapse formation and the expression of mature cell types (Figure 1). Importantly, brain organoids have shown functional maturation and consistent network activity that support studies in the context of human brain evolution and diseases.

## Brain Disease Modeling

**Figure 2:**
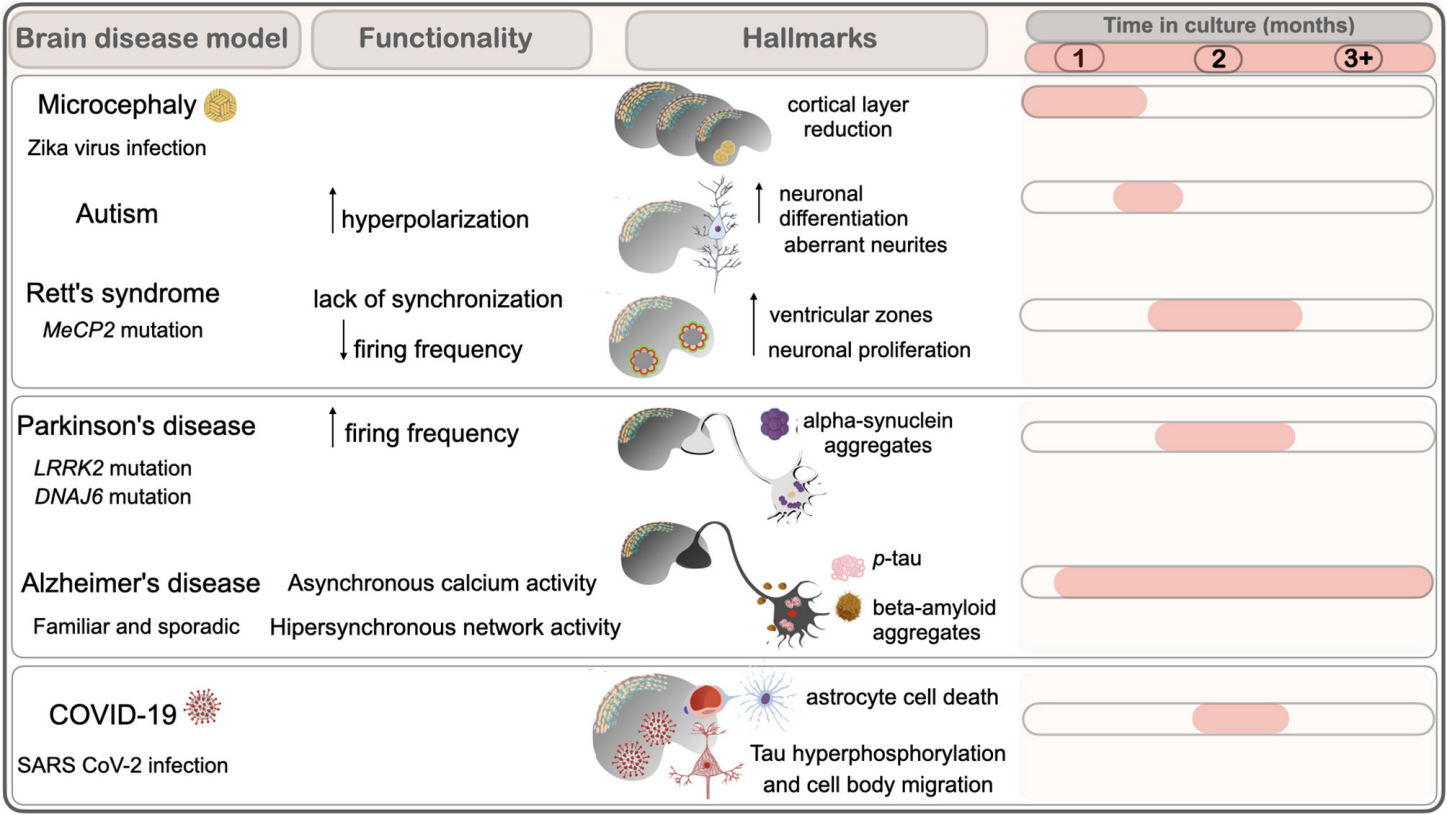
Brain organoids models display hallmarks of neurodevelopmental and neurodegenerative diseases. Time in culture (months) of hallmarks and functional alterations observed in organoids modeling brain disorders are depicted. Neurodevelopmental disorders include microcephaly by viral infection (Zika virus), Autism and Rett's syndrome. Neurodegenerative diseases include Parkinson's and Alzheimer's, and viral infection by SARS-CoV2.

Rodents have been heavily used in multiple studies, but limitations to replicate brain cytoarchitecture complexity (i.e., the cortical gyrification); formation of protein aggregates characteristic of neurodegenerative pathologies, and differential susceptibility to pathogens have hindered to address important aspects of developmental and neurodegenerative disorders; and pathogen invasion into the brain [reviewed in Gerakis and Hetz (2019); Marshall and Mason (2019)]. iPSC from patients containing mutations in causal genes and iPSC with mutations introduced by CRISPR/Cas9 have been used to produce brain organoid models for multiple diseases (Sidhaye and Knoblich, 2021). The possibility to address the complexity of cell types found in a tissue together with physiologically relevant phenotypes helped to elucidate mechanisms of disease in the human tissue, which were not contemplated before. Disease models based on brain organoid and relevant findings thereof are discussed in this section, as well as the hallmarks and disturbances in their functionality (Figure 2).

### Neurodevelopmental Disorders

#### Microcephaly

Primary microcephaly is characterized by a reduction of brain size caused by insufficient production of neural progenitors and neurons during fetal development (Jayaraman et al., 2018). Primary microcephaly was elegantly modeled in the 2013 whole-brain organoid protocol from Lancaster (Lancaster et al., 2013). In that work, iPSCs from patients with microcephaly formed smaller embryoid bodies and showed reduced neuroepithelial tissue at an early stage of organoid development (22-days) (Figure 2).

Inherited mutations or external perturbations can lead to microcephaly mainly by the disruption of cell division-related processes (Jean et al., 2020). Brain organoids display the positioning of radial glia-like cells in an orientation that resembles that of a ventricular zone facilitating the study of oriented cell divisions. This way, the pattern of symmetrical/asymmetrical divisions in humans could be studied *in vitro* (Lancaster et al., 2013). Symmetrical divisions account for the expansion of neural progenitors, whereas asymmetric divisions generate neurons. Increased asymmetric divisions anticipate the generation of neurons and result in reduction of cortical size (Matsuzaki and Shitamukai, 2015).

Mutations in genes involved in cell division were shown to produce organoids with reduced size at an early stage of development. CDK5RAP2 participates in cell division coordinating microtubule dynamics as it has microtubule-organizing centers. iPSCs from patients with CDK5RAP2 mutation were used to produce whole-brain organoids. Loss of function of CDK5RAP2 protein resulted in a decrease in symmetrical cell divisions, and reduction of neurogenic regions and premature neurogenesis was observed as a marked increase in the number of BrdU+/doublecortin (DCX)+ cells in patient organoids (Lancaster et al., 2013).

Mutation in the *Aspm* gene (abnormal spindle-like microcephaly-associated) is the most common cause of human primary microcephaly. iPSCs derived from a patient with a mutation in the Aspm gene formed smaller embryonic bodies compared to control. Reduction in neuroepithelial regions was observed in cortical organoids together with reduced staining for Pax6, Sox2, and ZO-1 as well as fewer proliferative Ki67+ and pH3+ cells. On day 65 of development (~2 months), Aspm mutant organoids showed disorganized lamination as seen by Tbr2 immunostaining. Calcium signaling could be observed at day 85 (2.8 months) of development and showed no differences in transient frequencies, but mutant organoids were less synchronized than controls, suggesting that those cells were not able to develop mature circuits (Li et al., 2017).

The future characterization of other mutations leading to microcephaly can amplify the knowledge about the developmental processes that guide brain size [reviewed in Gabriel et al. (2020)]. Also, it opens possibilities to investigate how the establishment of a neural progenitor pool has a role in circuit formation. Reduced organoid size in response to pathogens has also been described and will be discussed in the topic: organoids as a model to study infectious disease.

#### Rett Syndrome

Rett syndrome (RTT) is a rare neurodevelopmental disorder characterized by speech and motor disabilities, along with cognitive impairment, which start to appear after a period of normal development. Mutations in the methyl-CpG-binding protein 2 (MeCP2) gene are the cause of Rett syndrome, and the spectrum of severity can vary depending on the site of mutation. Since the MeCP2 gene is located in the X chromosome, female cells present a mosaic pattern of expression (Chahrour and Zoghbi, 2007).

Rett syndrome was studied using iPSC cells from patients carrying mutations in the MeCP2 gene. Neuronal cultures differentiated from patients' iPSCs allowed the characterization of an *in vitro* phenotype showing reduced synapses, altered calcium signaling, defective firing activity, and excitatory/inhibitory imbalance (Marchetto et al., 2010). MeCP2 is critical for the functioning of gabaergic neurons (Chao et al., 2010). Using neurons derived from iPSCs from RTT patients, it was possible to show that downregulation of a neuron-specific K+-Cl– cotransporter2 (KCC2) was the cause of GABA functional deficits in Rett neurons, impeding GABA functional switch (Tang et al., 2016). Because KCC2 starts to be expressed postnatally and increases throughout the lifespan, deficits in KCC could explain why the disease starts to manifest early in childhood and progressively increases severity with aging.

Studies addressing 3D whole-brain organoid models of RTT showed deficits in neurogenesis, with an increase of ventricular zones at 1.15 months (5 weeks) of development. Increased proliferation in detriment of neuronal generation was suggested as the cause of delayed development observed in 2D neuronal cultures (Mellios et al., 2018). MEA electrophysiology conducted in MECP2-KO cortical organoids showed decreased population spiking compared with the controls, which could be rescued with treatment with selected compounds from a drug screening (Trujillo et al., 2021). Region-specific organoids with cortical specialization and medial ganglionic eminence (MGE), a critical ventral brain domain producing cortical interneurons and related lineages (Xiang et al., 2017), were generated from hESC with MeCP2 mutation. Calcium signaling and depolarization-dependent c-FOS induction were analyzed in wt and MeCP2 mutant organoids and showed lack of synchronization of calcium surges and deficient c-Fos induction in MGE organoids. Synchronization of calcium surges was not affected in cortical organoids, although c-Fos induction was compromised, suggesting that cortical areas, isolatedly, are less sensitive to MeCP2 mutation. Transcriptional analysis of 2.4 months (74 days) organoids suggested that potential deficits in GABAergic activity seen in these organoids could be reversed by a drug affecting epigenetic pathways (Xiang et al., 2020).

Patient-derived female iPSCs carrying a MeCP2 mutation were used to generate lines with an isogenic control of the MeCP2 mutation. Dorsal organoids were generated and altered intracellular calcium dynamics were observed in neurons from dissociated organoids at 1.5 months (44–47 days) and 2.2 months (64–67 days). Alterations in neuronal activity were measured using patch-clamp at 1.5 and 2.6 months (81 days), revealing a reduction in the firing frequency and abortive-like action potential in mature MeCP2 mutant neurons. The fusion of region-specific organoids from MGE and cortex revealed defective interneuron migration in MeCP2 mutant assembloids at day 41 (1.3 months) (fusion of assembloids occurred at day 13) (Gomes et al., 2020a). In the future, it will be important to expand the analysis of RTT assembloids to address how dysfunction of interneurons caused by MeCP2 mutation can interfere with circuitry formation and cortical functioning.

#### Autism Spectrum Disorders

Autism spectrum disorder (ASD) is a frequent neurodevelopmental disorder characterized by impairments in social interaction, communication, and behavioral flexibility (DMS-5, American Psychiatric Association, 2013). The genetic determinants of ASD have been largely investigated, and due to the clinical heterogeneity of ASD, understanding how key genes play a role in the establishment of ASD is a challenging task. A recent large-scale exome sequencing study identified 102 risk genes related to autism. Most risk genes are expressed in early brain development and have a role in the regulation of gene expression or in neuronal communication (Satterstrom et al., 2020).

CDH8 (chromodomain helicase DNA-binding protein 8) is a chromatin remodeling factor, which was identified as a top candidate gene for ASD from independent exome-sequencing studies and is also involved in schizophrenia and intellectual disabilities (Neale et al., 2012; O'Roak et al., 2012; Talkowski et al., 2012; Bernier et al., 2014; Krumm et al., 2014; McCarthy et al., 2014). The investigation of the transcriptional profile of neural lineages upon CDH8 knockdown or knockout has been used to understand which genes and pathways are affected in ASD. The sh-RNA knockdown of CDH8 in neural progenitor cells and a neuroblastoma cell line affected genes involved in neurodevelopment and altered the expression of genes related to autism (Bernier et al., 2014; Sugathan et al., 2014; Cotney et al., 2015). CDH8 knockout in an iPSC line was introduced using the CRISPR-Cas9 technology, generating a mutant cell line that could be compared with its isogenic control (Wang et al., 2015). Neural progenitor cells and a mixed neuronal culture positive for markers from distinct brain regions were differentiated from mutant cells and isogenic controls, and regulated genes were involved in cell adhesion, neuron differentiation, neuron projection, synaptic transmission, axonal guidance signaling, and WNT/β-catenin and PTEN signaling (Wang et al., 2015).

Brain organoids were generated from CHDH8^−/−^, two heterozygous lines, and two isogenic controls (Wang et al., 2017). Transcriptional analysis at 1.6 months (day 50) revealed a significant overlap (~50%) to genes regulated in CHDH8^−/−^ neural progenitor cells and neurons. DLX6-AS1 and DLX1, which regulate gabaergic neuron development, are among the top regulated genes in CHDH8^−/−^ brain organoids. DLX6-AS1 was also one of the top genes found in a transcriptome study of brain organoids from idiopathic ASD patients, which were analyzed on days 11 and 31 *in vitro* (Mariani et al., 2015). Other genes involved in brain development, and previously implicated in ASD, were found to be regulated in CHDH8^−/−^ and idiopathic ASD, including FZD8, PAX6, SLC1A3, EOMES (TBR2), and MPPED1 (Mariani et al., 2015; Wang et al., 2017), showing that distinct genetic alterations can lead to convergent alterations in gene expression.

The transcriptional analysis of brain organoids from ASD probands revealed alterations in cell cycle genes. The cell cycle in ASD was reduced in length in comparison to controls, resulting in increased neuronal differentiation and overproduction of neurons, and the ratio between glutamatergic and gabaergic neurons was altered (Mariani et al., 2015). Sodium currents in ASD neurons from brain organoids tended to inactivate at more hyperpolarized membrane potentials than in controls. Half maximal amplitude values in the range −72 to −65 mV suggested increased expression of the Nav1.1 isoform, which is preferentially expressed in GABAergic interneurons (Ogiwara et al., 2007) and consistent with a greater proportion of GABAergic neurons in ASD cortical organoids. Importantly, FOXG1 was upregulated in idiopathic ASD, but not CHDH8-/- brain organoids, and ASD phenotype could be reverted by FOXG1 knockdown (Mariani et al., 2015).

The importance of early steps in development to the establishment of ASD phenotypes was demonstrated in brain organoids from patients with ASD. Quantitative assessment of neuronal branching in 6-week organoids showed aberrantly complex neurite structures in ASD neurons when compared to controls. Notably, circumventing the neural stem cell step of development by using a directed differentiation protocol avoided the ASD phenotype (Schafer et al., 2019). This study highlighted the importance of models that recapitulate early stages of development to the comprehension of ASD phenotype.

Moreover, ASD risk genes are enriched in excitatory and inhibitory neuronal lineages, and a combination of gene expression profiles could promote the excitatory-inhibitory imbalance underlying ASD (Satterstrom et al., 2020). It would be interesting, in the future, to address how risk genes can affect brain organoid circuitry at the electrophysiological level and how it correlates to ASD phenotypes.

### Viral Diseases

Multiple aspects of viral infection in the brain can be reproduced using brain organoids. Brain damage and viral replication in the brain tissue were demonstrated, eliciting the investigation of transcriptional networks, types of affected cells, and the evaluation of immune response. We highlight the work in the neurotrophic zika virus (ZIKV) and in the new coronavirus SARS-CoV-2, and how brain organoids helped to elucidate aspects of viral infection in the brain tissue.

#### Zika Virus

ZIKV is a mosquito-borne flavivirus that became a global health concern after the connection between infection during pregnancy and the occurrence of microcephaly in children born from infected mothers was established (www.who.int). ZIKV experimental infection in brain organoids induced cell death and reduced proliferation. Also, a decrease in neuronal cell-layer volume and in organoid size resembling microcephaly phenotypes was observed following infection (Cugola et al., 2016; Dang et al., 2016; Garcez et al., 2016; Qian et al., 2016). Moreover, ZIKV infection led to mitochondrial failure, oxidative stress, and DNA damage in human iPSC-derived astrocytes and mitochondrial damage in brain organoids (Ledur et al., 2020).

The molecular basis of ZIKV infection was further explored in proteomic and transcriptomic analysis of ZIKV infected neurospheres, a 3D model of neural culture lacking cortical layer organization, revealing alterations in molecular pathways regulating cell cycle, neuronal differentiation and innate immune response (Garcez et al., 2017). Accordingly, upregulation of the innate immune receptor Toll-Like-Receptor 3 (TLR3) was shown after ZIKV infection of human organoids at early days *in vitro* (15 days), and inhibition of TLR3 reduced the phenotypic effects of ZIKV infection (Dang et al., 2016).

Infection of human brain organoids has been used for the screening of compounds and drug repurposing to treat infection (Xu et al., 2016, 2019; Watanabe et al., 2017; Pettke et al., 2020). Strikingly, brain organoids were used by our group to establish a connection between ZIKV infection and the regional increase in the cases of microcephaly in the northeast of Brazil. Analysis of the quality of water in reservoirs revealed the presence of saxitoxin in a period concomitant to the appearance of microcephaly cases suggesting that STX and ZIKV could act as a co-insult, aggravating that condition. We showed that treatment with STX enhanced ZIKV phenotype in brain organoids, raising awareness about the impact of water quality in viral infection (Pedrosa et al., 2020).

#### SARS-CoV-2

The strike of COVID-19 pandemic fuelled research in the organoid field to try to understand the neurological symptoms associated with the disease. Although COVID-19 mainly affects the respiratory tract, neurological symptoms are also present in 30–60% of patients, including paresthesia, altered consciousness, and headache (De Felice et al., 2020; Wang et al., 2020). COVID-19 increases the risk of stroke by 7-fold, and 2–6% of patients develop cerebrovascular disease (Fifi and Mocco, 2020). Also, encephalopathy, encephalitis and Guillain-Barré syndrome appear as less frequent neurological manifestations of COVID-19 (Ellul et al., 2020; Mao et al., 2020).

Experimental SARS-CoV-2 infection in brain organoids helped to elucidate types of cells affected by this virus. Cortical organoids at 2 weeks and 1 month were infected with SARS-CoV-2 and fewer SARS-CoV-2 positive cells were identified at 2 weeks than 2 months, revealing a preference for the infection of mature neurons. Also, neurons from brain organoids in an air-liquid interface culture, plated at 2 months and migrated for 2 weeks, were positive for SARS-CoV-2. In all systems analyzed, infection was not shown to be productive. Two-month-old organoids positive for SARS-CoV-2 exhibited an altered Tau localization pattern from axons to soma, Tau hyperphosphorylation, and apparent neuronal death (Ramani et al., 2020). Neuronal infection was shown by other groups (Mesci et al., 2020; Song et al., 2021) and productive infection was observed using high multiplicity of infections (MOI 10). However, the comparison of the conditions used to infect neurosphere cultures using a different SARS-CoV-2 isolate could not confirm productive infection, suggesting that differences between strains can affect neurotropism (Pedrosa et al., 2021). SARS-CoV-2 infection could be prevented by blocking ACE2 with antibodies, and by using cerebrospinal fluid from a COVID-19 patient (Song et al., 2021). Analysis of ACE2 expression in the brain revealed much higher expression in the choroid plexus than in the cortex (Pellegrini et al., 2020), and a much higher SARS-CoV-2 tropism to choroid plexus was identified, but little to no infection of neurons or glia was shown (Jacob et al., 2020; Pellegrini et al., 2020). Infection of choroid plexus organoids was demonstrated (Jacob et al., 2020; Pellegrini et al., 2020) and cell death and transcriptional dysregulation indicative of an inflammatory response and cellular function deficits was seen (Jacob et al., 2020). Leakage in choroid plexus organoids was observed, suggesting a functional loss in the barrier function of this tissue *in vitro* (Pellegrini et al., 2020), which could make brain tissue vulnerable to the entry of pathogens and inflammatory molecules. These findings were corroborated with *post-mortem* studies showing SARS-CoV-2 infection in the choroid plexus (Gomes et al., 2020b).

Recently, assembloids constituted by pericytes and cortical organoids were shown to facilitate the infection of astrocytes by SARS-CoV-2. Astrocytic cell death was shown along with increased cell stress and inflammation (Wang et al., 2021). Given that COVID-19 severely damages the brain vascular system (Lee et al., 2021a), vascularized organoids could contribute to understanding the role of the vascular tissue damage in brain infection. Additionally, since there is increasing evidence of neurological and neuropsychiatric complications of COVID-19 (Varatharaj et al., 2020; Ding et al., 2021), it would be interesting to study SARS-CoV-2 infection in models to replicate diseases that may lead individuals most vulnerable to neurological COVID-19 sequelae.

### Neurodegenerative Disorders

Parkinson's Disease (PD) and Alzheimer's Disease (AD) are the most common progressive, age-related neurodegenerative conditions. Distinct brain areas are affected by PD and AD. PD affects mainly the striatum, resulting in motor impairment. The entorhinal cortex is the first to be affected in AD, resulting in cognitive decline. Importantly, protein aggregation is a pathological hallmark of these diseases, which could be replicated in *in vitro* 3D models. Models of AD and PD and their implications to the pathophysiological mechanisms of these diseases are discussed below. Few studies, which will not be addressed in this review, used brain organoids to investigate other important neurodegenerative diseases, including Huntington's Disease, Amyotrophic Lateral Sclerosis (ALS), Motor Neuron Disease (MND), and Frontotemporal Dementia (Venkataraman et al., 2020; Sidhaye and Knoblich, 2021).

#### Alzheimer's Disease

AD is characterized by progressive neurodegeneration and memory loss and is the most prevalent cause of dementia accounting for 60–80% of cases. The presence of senile plaques, constituted by amyloid-β peptides (Aβ) and neurofibrillary tangles (NFTs), formed by phosphorylated tau, are two major neuropathological hallmarks in AD which often precede the onset of symptomatic dementia by decades (Scheltens et al., 2016). In addition, brain stress signaling and glucose metabolism are altered in AD along with a pro-inflammatory profile contributing to AD pathophysiology (Clarke et al., 2015).

Familial AD (FAD) accounts for <5% of AD cases and appears at an early onset (~40 years old) when compared to sporadic AD (SAD), which accounts for the majority of cases and develops later in life (>60 years old) (Paul Johns, 2014). FAD is caused by variants in the presenilin-1 (PSEN1), presenilin-2 (PSEN2), or amyloid precursor protein (APP) genes, whereas SAD is associated with multiple genetic polymorphisms. Known causal variants include the ε4 haplotype in APOE, the strongest genetic risk factor for late-onset AD (Lanoiselée et al., 2017; Schwartzentruber et al., 2021). Also, because APP is located in C21, a connection between gene dosage and AD is seen in 21 trisomy, and Down syndrome adults have increased risk to develop AD and show neuropathological changes of AD at age 40 (Wiseman et al., 2015; Lott and Head, 2019).

AD pathology could be replicated in iPSC-derived neurons from AD patients showing an increase in Aβ, phosphorylated tau, and cellular stress markers (Yagi et al., 2011; Kondo et al., 2013; Muratore et al., 2014). The use of brain organoids allowed for the detection of extracellular protein aggregation in a complex tissue. Brain organoids obtained from patients with FAD with *APP* duplication and *PSEN1* mutation and from individuals with Down syndrome could replicate Aβ accumulation and tau pathology at 1 month, 2 months, and after 3 months in culture (Figure 2) (Raja et al., 2016; Gonzalez et al., 2018; Alić et al., 2020). Investigation of such models showed that APOE4 allele aggravated tau pathology in brain organoids from patients at 3 months *in vitro*. In addition, the authors showed that the isogenic conversion of APOE4 to APOE3 allele attenuated the AD-related phenotypes (Zhao et al., 2020).

Neuronal hyperactivity is observed in patients with FAD and SAD (AD), leading to non-convulsive epileptic discharges (Vossel et al., 2013; Palop and Mucke, 2016; Lam et al., 2017). Increased calcium transients could be observed in brain organoids from FAD patients (Park et al., 2018) and asynchronous calcium and enhanced neuronal hyperactivity were observed at 40+3 weeks (Yin and VanDongen, 2021). AD neuronal activity in 2-month-old cerebral organoids from FAD patients plated in MEA displayed an increase in action potential firing rate compared to WT organoids and also showed increased VGLUT1 and decreased VGAT staining (Ghatak et al., 2019). Following these observations, a report from the same group showed hypersynchronous network activity in AD organoids, which could be reverted by the dual-allosteric NMDAR antagonist NitroSynapsin, but not by memantine, a drug approved by the FDA for AD treatment (Ghatak et al., 2020).

The role of microglia in AD pathology was studied in co-culture experiments in which microglia-like cells were co-cultured with brain organoids with APP mutation. APOE3- or APOE4 microglia-like cells were added to 2-month-old AD organoids that displayed Aβ aggregates. After 1 month of co-culture, APOE4 allele exhibited more extracellular Aβ aggregates than those co-cultured with APOE3 microglia-like cells, showing that APOE4 negatively impacts microglial function with a poor ability to clear extracellular Aβ from AD brains, and possibly influencing the brain inflammatory profile (Lin et al., 2018).

So far, most of the contributions from brain organoids to the understanding of AD have focused on FAD. Although FAD replicated multiple aspects of SAD with the possible advantage of being detected at earlier times in brain cultivation, it would be important to understand how models for SAD manifest Alzheimer pathology *in vitro*. iPSC and CRISPR/CAs-9 technology would allow for the analysis of candidate risk genes and their combinations, possibly setting up a model for SAD (Schwartzentruber et al., 2021). Additionally, metabolic imbalance and the role of immune response in AD brain organoids, both of which are current therapeutic targets for AD (Clarke et al., 2015) have been poorly explored. These aspects should be taken into account when using brain organoids for drug discovery.

#### Parkinson's Disease

PD is characterized by the progressive development of movement impairments, and other non-motor symptoms caused by loss of dopaminergic neurons from substantia nigra (SN) in the midbrain. A pathological hallmark of PD is α-synuclein deposition in Lewy bodies. Accumulation of α-synuclein generates synaptic dysfunction which leads to neurodegeneration (Schulz-Schaeffer, 2010; Stefanis, 2012). A mutation in the SNCA gene coding for α-synuclein was the first to be related to PD. Today, multiple risk genes are associated with PD, and missense mutations in the leucine-rich repeat kinase 2 (LRRK2) gene locus are the most common known causes of late-onset familial and sporadic PD (Di Fonzo et al., 2005; Nalls et al., 2019).

Advances in the development of midbrain organoids allowed to test PD in cultured human tissue (Jo et al., 2016; Smits et al., 2019). Sporadic Parkinson's Disease was modeled in midbrain organoids generated from patients carrying a mutation in the LRRK2 gene and compared to isogenic controls. Control midbrain organoids showed a progressive increase in dopaminergic neurons over time (from day 10 to 70), whereas, in patient-derived midbrain organoids, the development of dopaminergic neurons was impaired. Patient-derived midbrain organoids also showed reduced complexity of midbrain dopaminergic neurons (Smits et al., 2019). Reduced astrocytic activity was shown in 35-day old (1.15 month) midbrain PD organoids, which may be associated with neurodegeneration (Kano et al., 2020). Transcriptomic analysis of brain organoids (at 1.5 and 2 months in culture) derived from iPSC in which LRRK2 mutation was introduced using CRISPR/Cas9 revealed the upregulation of a thioredoxin-interacting protein (TXNIP), which was previously reported to be associated with lysosomal dysfunction in α-synuclein-overexpressed cultures. The knockdown of *TXNIP* suppressed the aggregation of phosphorylated α-synuclein in lysosomes (Kim et al., 2019). This was the first study to provide a direct functional connection between α-synuclein and *LRRK2* through *TXNIP* endorsing the power of brain organoid technology to investigate molecular mechanisms of PD.

A recent publication described a model of early-onset PD in which a mutation in *DNAJ6*, described in familial juvenile parkinsonism, was introduced in hESC using CRISPR-Cas9 (Wulansari et al., 2021). *DNAJ6* mutant midbrain organoids showed defective wnt signaling at day 15, showing that this mutation has an impact on early neurodevelopment. Less dopaminergic neurons were found, and dopaminergic neuron degeneration was observed in midbrain organoids with *DNAJ6* mutation (Wulansari et al., 2021), similarly to previous PD models (Smits et al., 2019). Increased reactive oxygen species (ROS), lower levels of dopamine release, increased α-syn oligomers, mitochondrial and autolysosomal dysfunctions were also observed in mutant midbrain organoids (~2 months) (Figure 2) (Wulansari et al., 2021). Mutant midbrain organoids showed an increase in firing frequencies at 2.6 months (DIV80) (Wulansari et al., 2021). The authors correlate this finding with an increase of intrinsic pacemaker frequency, a neuronal characteristic in progressive PD, which is caused by oxidative impairment of A-type Kv4.3 potassium channels (Subramaniam et al., 2014).

The development of non-invasive methods to address organoid-models for neurodegeneration are paramount to measure maturity and pathological features over time. An example of such technology is a pathology imprinted electrode that can detect alpha-synuclein at femtogram levels, which was used to measure alpha-synuclein in the culture medium of human brain organoids generated from normal and idiopathic PD patients (Lee et al., 2021b).

## Conclusions

Human organoid technology is undoubtedly an advance for the understanding of early human brain development, particularly when compared to animal models that were the best alternative to study the brain prior to iPS technology. While brain organoids offer a range of possibilities, researchers need to carefully consider: (1) the specific organoid protocol to use in order to have a model that is enriched in the cell type(s) that is (are) most relevant to what they wish to study; (2) time in culture—the age of the organoid—to allow for the target cell types to differentiate; (3) the proper time in culture to allow for cell maturity and full functionality. This way, if all 3 items mentioned are thoroughly considered, brain organoids can be successfully applied to brain disease modeling. In the context of neurological disorders, iPSC-derived from healthy subjects, patients carrying mutations, or genomic editing offer a variety of phenotypes to be studied. With advances in obtaining brain organoids, the prototype of a room for growing and maturing brain organoids could be conceived, similarly as cell culture or animal facilities in research institutes. In the near future, we hope that the establishment of robust, reproducible methodological settings as well as functional assessments at accessible costs will propel science in the brain organoid field.

### Perspectives

The evolving field of brain organoids allowed the investigation of human diseases, leading to the molecular understanding of human brain tissue that was otherwise inaccessible. While reproducibility is a challenge, current initiatives to set standards through the comparison across multiple labs and over many replicates are ongoing. The Organoid Cell Atlas project (https://hca-organoid.eu) aims to compare organoids' cell type composition with that of the developing human brain using single-cell sequencing (Bock et al., 2021). With that, we will be able to appreciate information about the developmental trajectory of brain organoids, potentially expanding this knowledge to access models of brain disorders.

The application of novel analytical technologies is key to the improvement of brain organoids as a model. Following single-cell sequencing, single-cell proteomics is an emerging technology that can be applied to disclose biological processes taking place during development, as it can reveal effective protein amounts in cells and post translational modifications which are relevant for protein function (Goto-Silva and Junqueira, 2021). CRISPR technology is and will continue to be a major technology in the field, allowing us to explore the manipulation of a few genes in an isogenic background and pinpoint allele-specific phenotypes. On top of that, multiple molecular biology-based tools can be introduced with CRISPR editing to allow deep functional exploration of cell lineages in brain organoids (Esk et al., 2020). This tool may also incorporate genetic manipulations of protein domains, dominant active, and tagged forms of proteins. Remarkably, the spatial and temporal precision afforded by optogenetics has recently been proven useful for assessing and controlling the activity of neural circuits in brain organoids (Andersen et al., 2020).

Lack of throughput is still a limitation in current organoid protocols. Implementation of high throughput, microfluidic and compartmentalized platforms may allow handling cultures from multiple individuals (Renner et al., 2020; Velasco et al., 2020). This will be an important step toward the understanding on how developmental trajectories are established across subjects. Concurrently, multifactorial neurological diseases such as neuropsychiatric disorders could be addressed, revealing also how neural activity is affected in such conditions.

Filling the gaps between *in vitro* findings and patient phenotypes is among the major challenges in having brain organoids to model disease. The connections among distinct brain regions reproduced in brain assembloids can help us understand alterations in the connectivity of neural networks in different conditions. A robust model to address connectivity and neuron activity can bring *in vitro* research closer to the neuroimaging field so that we can start translating connectivity measurements from human brains to human brain organoids.

## Author Contributions

LOP and PFL drafted the outline. LOP, PFL, and LG-S wrote the manuscript. LOP prepared the figures. The manuscript was then reviewed and edited by SKR. All authors have approved the final version of the manuscript.

## Conflict of Interest

The authors declare that the research was conducted in the absence of any commercial or financial relationships that could be construed as a potential conflict of interest.

## Publisher's Note

All claims expressed in this article are solely those of the authors and do not necessarily represent those of their affiliated organizations, or those of the publisher, the editors and the reviewers. Any product that may be evaluated in this article, or claim that may be made by its manufacturer, is not guaranteed or endorsed by the publisher.
